# The role of glutathione S-transferases in human disease pathogenesis and their current inhibitors

**DOI:** 10.1016/j.gendis.2024.101482

**Published:** 2024-12-05

**Authors:** Sulaiman Mohammad Alnasser

**Affiliations:** Department of Pharmacology and Toxicology, College of Pharmacy, Qassim University, Qassim 51452, Saudi Arabia

**Keywords:** Drug metabolism, Environmental toxins, Genetic variations, Glutathione S-transferases, Oxidative stress

## Abstract

Glutathione S-transferases (GSTs) are a family of enzymes detoxifying various harmful compounds by conjugating them with glutathione. While primarily beneficial, dysregulation of GST activity or specific isoforms can contribute to disease pathogenesis. The intricate balance of detoxification processes regulated by GSTs is pivotal in cellular homeostasis, whereby dysregulation in these mechanisms can have profound implications for human health. Certain GSTs neutralize carcinogens, shielding cells and potentially preventing tumorigenesis. Polymorphisms in specific GSTs may result in the accumulation of toxic metabolites, exacerbating oxidative stress, inflammation, and DNA damage, notably observed in neurodegenerative diseases like Parkinson's disease. They can also modulate signaling pathways involved in cell proliferation, survival, and apoptosis, with aberrant activity potentially contributing to uncontrolled cell growth and resistance to cell death, thus promoting cancer development. They may also contribute to autoimmune diseases and chronic inflammatory conditions. This knowledge is useful for designing therapeutic interventions and understanding chemoresistance due to GST polymorphisms. A variety of GST inhibitors have been developed and investigated, with researchers actively working on new inhibitors aimed at preventing off-target effects. By leveraging knowledge of the involvement of specific GST isoforms in disease pathogenesis across different populations, more effective and targeted therapeutics can be designed to enhance patient care and improve treatment outcomes.

## Introduction

Glutathione S-transferases (GSTs), formerly known as ligandins, belong to a class of prokaryotic and eukaryotic phase II metabolic isoenzyme.^1^ These eight dimeric enzymes are categorized as alpha (A), kappa (K), mu (M), omega (O), pi (*p*), sigma (S), theta (T), and zeta (Z) based on their amino acid sequences and substrate specificity.^2^ Cytosolic dimeric proteins, which typically have molecular weights of approximately 50 kDa, make up the great bulk of GSTs. They can assist conjugation of the reduced glutathione (GSH) with xenobiotic substrates, aiming to detoxify them^3^ and play a vital role in maintaining cellular equilibrium. To produce the reduced form of GSH conjugates, reduced GSH must bind to reactive electrophiles generated during cytochrome P450 metabolism. This process underscores the predominant cytoprotective function of GSTs. The subsequent GSH conjugates are either delivered to the kidney or eliminated by bile.^4^ There, γ-glutamyl transpeptidase catalyzes the cleavage of the γ-glutamyl moiety, dipeptidase cleaves the glycine, and N-acetylation modifies the cysteine.^5^

They are crucial players in cell signaling^6^ and while detoxifying, specific GST classes, particularly pi and mu, directly interact with signaling molecules like c-Jun N-terminal kinase 1 (JNK1) and apoptosis signal-regulating kinase (ASK1).^7^ This interaction directly modulates the mitogen-activated protein (MAP) kinase pathway, a central hub controlling cell survival and death signals.^5^ GSTs influence this pathway through two key mechanisms: post-translational modifications of other proteins, adding complexity to the signaling cascade, and direct protein interactions with JNK1 and ASK1.^8^ In response to varied extracellular stimuli, GST gene expression has been demonstrated to be co-regulated either transcriptionally or post-transcriptionally. Cytoprotective agents stimulate the expression of GST while simultaneously activating the PI3K-Akt/ERK-RSK1-mTOR pathways. This activates transcription factors and promotes cell viability.^9^ Common substrates for GST-mediated glutathionylation encompass protein disulfide isomerase, peroxiredoxin-VI, and P53 ^5^. In addition to their detoxification function, GSTs play a significant role in a diverse array of biological processes. They participate in the metabolism of endogenous compounds, including hormones, fatty acids, and prostaglandins. Furthermore, GSTs are implicated in drug metabolism and resistance.^10^ They can metabolize certain drugs and chemotherapeutic agents, affecting their efficacy and toxicity. Membrane-bound GS-X pump transports antineoplastic drugs adhering to GSH, thereby conferring resistance to chemotherapy in cancer cells.^2^ Additionally, overexpression of certain GST isoforms is concomitant to drug resistance in cancer cells, making them potential targets for overcoming chemotherapy resistance.^11^ Overall, GSTs are versatile enzymes with diverse functions in cellular detoxification, metabolism, signaling, and drug response. Understanding their role in these processes is essential for elucidating their contribution to health and disease and for developing strategies for therapeutic intervention.

## Classification based on subcellular location

GSTs can be categorized into cytosolic, mitochondrial, microsomal, or membrane-associated isoforms, based on their sub-cellular location.

### Cytosolic GSTs

These GSTs are primarily found in the cytoplasm of cells. They play a vital role in detoxification processes by catalyzing the conjugation of GSH with various electrophilic compounds. Mammalian cytosolic GSTs consist of 199–244 amino acid-long subunits that are all dimeric. Mammalian species have been identified to possess seven classes of cytosolic GSTs, namely alpha, mu, pi, sigma, theta, omega, and zeta, based on similarities in amino acid sequences. It has been discovered that non-mammalian species include other classes of cytosolic GST, including the “U" class, phi, tau, epsilon, beta, and delta. Cytosolic GST isoenzymes within a class typically exhibit more than 40% identity in rodents, whereas the identity between classes in humans is generally less than 25%.^12^ There are approximately 16 cytosolic GST subunits. The ability of members within the alpha and mu classes to form heterodimers results in a significantly larger number of isoenzymes being produced. The cytoplasm of the cell contains the vast majority of cytosolic GST isoenzymes. On the other hand, mouse GSTM1-1 and human and mouse alpha-class GSTA4-4 can bind to membranes and mitochondria. When it comes to GSTA4-4, this means that the transferase must be phosphorylated, and the Hsp70 chaperone is necessary for targeting. Apart from these GST classes, the crystal structure of elongation factor 1Bγ and chloride intracellular channels is identical to that of cytosolic GST. It is yet unknown whether other proteins, such as ganglioside-induced differentiation-associated protein-1, actually occupy the GST fold.

### Mitochondrial GSTs

These GSTs are localized within the mitochondria, the cellular organelles responsible for metabolism and energy production. They are involved in reactive oxygen species (such as superoxide anions, hydrogen peroxide, and hydroxyl radicals) and lipid peroxidation product (such as malondialdehyde, 4-hydroxynonenal, F2-isoprostane, leukotriene) detoxification generated during mitochondrial respiration. They contribute to the maintenance of mitochondrial function and cell viability by scavenging free radicals or safeguarding them through enzymatic reactions. The dimeric kappa GST isoenzymes found in the mitochondria of mammals are made up of 226 amino acid subunits. Humans, rats, and mice all have a single kappa GST. The mitochondrial GST has been shown through molecular cloning and crystallography to be a unique form of transferase. The three-dimensional structure of kappa exhibits similarities with prokaryotic disulfide-bond-forming DsbA and TcpG oxidoreductases, as well as bacterial 2-hydroxychromene-2-carboxylate isomerase. Unlike any of the cytosolic GST isoenzymes, kappa functions as a GSH-dependent oxidoreductase that catalyzes the conversion of 2-hydroxy-chromene-2-carboxylate to trans-O-hydroxy benzylidene pyruvate. This discovery has shed new light on the structural diversity of GSTs.^13^ GST class kappa has demonstrated the ability to reduce CuOOH and (S)-15-hydroperoxy-5,8,11,13-eicosatetraenoic acid. Additionally, it exhibits significant activity towards aryl halides, such as 1-chloro-2,4-dinitrobenzene. Due to its resemblance to 2-hydroxychromene-2-carboxylate isomerase, GST kappa is capable of metabolizing aromatic hydrocarbons, such as naphthalene.^14^

### Microsomal GSTs

Microsomal glutathione transferases (MGSTs), including isoform MGST1, MGST2, and MGST3 are also referred to as membrane-associated GSTs, are linked with the endoplasmic reticulum and other membrane-bound organelles.^15^ They are integral players in the metabolism of lipid-derived electrophiles and xenobiotics, encompassing various drugs and environmental toxins. Moreover, MGSTs contribute significantly to the biotransformation of lipophilic compounds, thereby regulating cellular lipid homeostasis. In 2006, the first structure of MGST was elucidated,^16^ focusing on MGST1, revealing a homotrimeric arrangement with active sites located at subunit interfaces. GSH enters the active site of MGST1 from the cytosol and penetrates deeper within the protein. This allows the thiolate anion to access the hydrophobic binding site exposed to the membrane, thereby aiding in the reduction of membrane-bound phospholipid hydroperoxides and the conjugation of additional hydrophobic electrophiles.^17^^,^^18^ Apart from being induced by oxidative stress, MGST1 can be directly activated via covalent modification by reactive intermediates. MGST2 has a pivotal role in intracrine signaling of endoplasmic reticulum, oxidative damage control, and cell death by producing leukotriene C4.^19^ MGST2 trimer structures limit catalysis to one active site at a time, and the biconical central pore modulates hydrophobicity, regulating solvent influx to optimize reaction conditions at the active site.^20^ Studies have also revealed that MGST2 catalyzes the activation of GSH, resulting in the formation of a thiolate essential for both GSH peroxidase activity and GSH conjugation reactions with electrophilic substrates such as 1-chloro-2,4-dinitrobenzene.^12^ On the other hand, MGST3 facilitates the conjugation of leukotriene A4 with reduced GSH, yielding leukotriene C4.^21^ Additionally, this enzyme exhibits GSH-dependent peroxidase activity towards lipid hydroperoxides.^22^

## Structure

**Figure 1:**
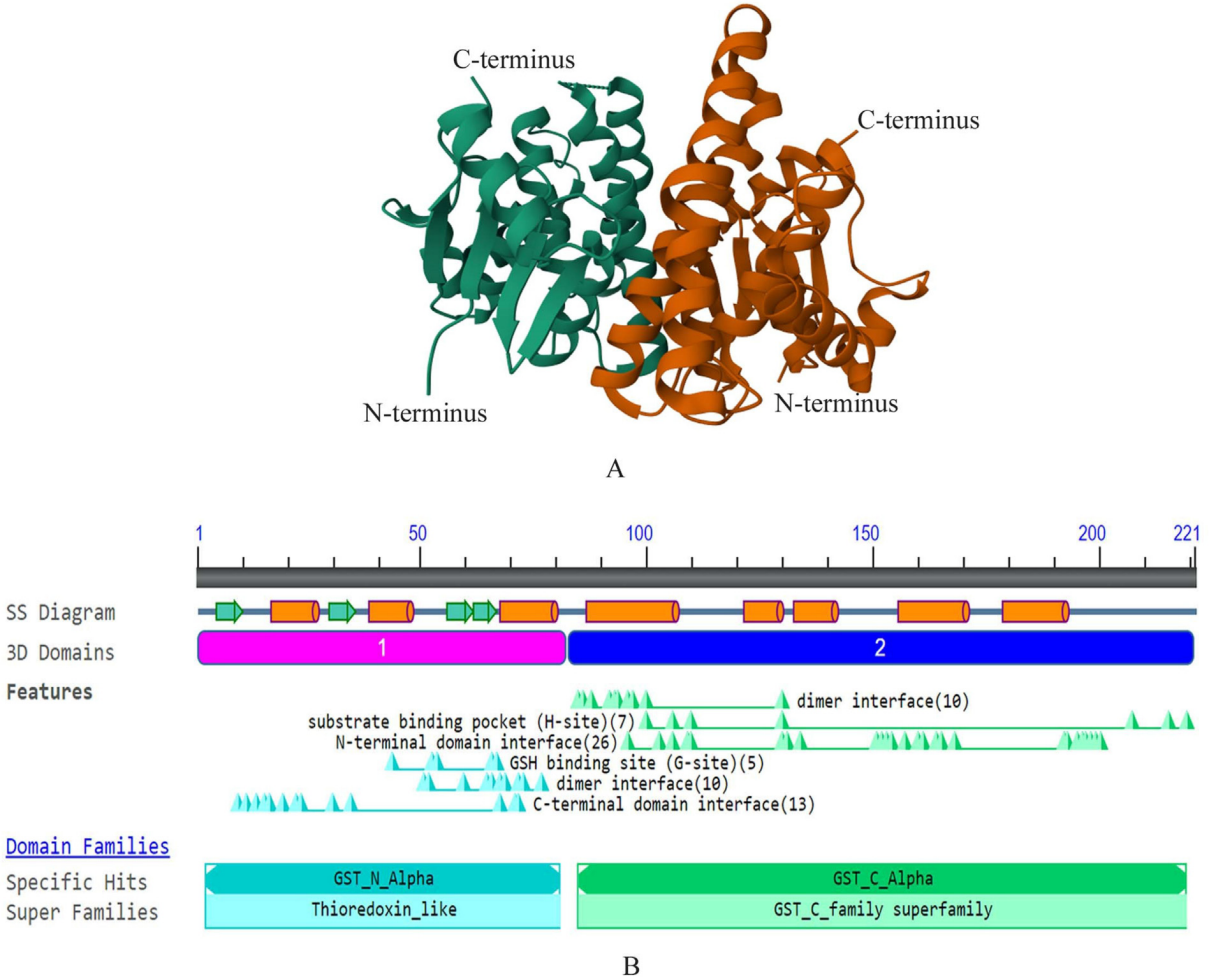
Structure and features of the GSTA1 protein **(A)** Three-dimensional dimeric structure of the human GST (PDB ID:1K3O), colored by chains A (green) and B (brown). N and C terminals are labeled by “N-" and “C-", respectively. **(B)** SS depicts secondary stricture. Helices are shown in orange barrels and strands in green arrows. Two major domains, the N-terminal TRX-fold domain, and the C-terminal alpha-helical domain are present, with an active site located in a cleft between the two domains. GSTA1, glutathione S-transferase alpha 1; GST, glutathione S-transferase; GSH, glutathione.

GSTs, owing to their pivotal role in xenobiotic metabolism, were among the first cytosolic proteins to have their structure elucidated, with the porcine GST Pi 1 (GSTP1) being the maiden member to be structurally characterized. The GST structure is characterized by a conserved catalytic domain, which is essential for their enzymatic activity. Catalytic GSTs are homodimers in mammals and have similar tertiary structures.^23^ The catalytic domain consists of approximately 200–250 amino acids and is responsible for binding both GSH and various electrophilic substrates. It also comprises key residues that facilitate the GSH-based nucleophilic attack on the electrophilic substrate, leading to the formation of a thioether bond and the subsequent detoxification of the substrate. The N- and the C-terminal domains comprise the fundamental protein fold, common to all major members of the GST family (Fig. 1). The β-α-β-α-β-α motif constitutes the N-terminal domain and makes up approximately one-third of the protein structure. The most conserved motif in this domain, referred to as the G-site, is β-β-α. It identifies the γ-glutamyl portion of GSH and serves at the site for binding to GSH.^12^ N- and C-terminal domains contribute to protein stability, subcellular localization, or protein–protein interactions.

Remarkably, all cytosolic and mitochondrial GSTs share a proline residue located at the N-terminus of the β3 strand. Proline interacts through hydrogen bonds with the GSH-cysteinyl moiety's backbone amine group. Two main subgroups have been identified by a global analysis of the sequence and structural similarities of GST proteins: Firstly, there are tyrosine-type GSTs (γ-GSTs), which employ tyrosine to activate GSH. Secondly, there are S/C-GSTs, which interact with GSH using serine (or cysteine), as observed in GST omega (GSTO).^24^

The remaining portion, constituting two-thirds of the protein structure, is composed of the distinct all-α-helical region, which forms the C-terminal domain of GSTs. The hydrophobic substrates attach to the H-site, a gap that exists between the N- and C-terminus domains. The shape and chemical makeup of the H-site varies significantly between GST classes, in contrast to the G-site. The substrate selectivity of different GST isozymes is also dependent on the variation in H-site structure.^25^ GSTs exhibit structural diversity among different isoforms and across species. This structural diversity allows GSTs to recognize and bind to a broad range of substrates, encompassing both endogenous compounds like hormones and prostaglandins and exogenous toxins like environmental pollutants and carcinogens.

## Functions of GSTs

### Detoxification

**Figure 2:**
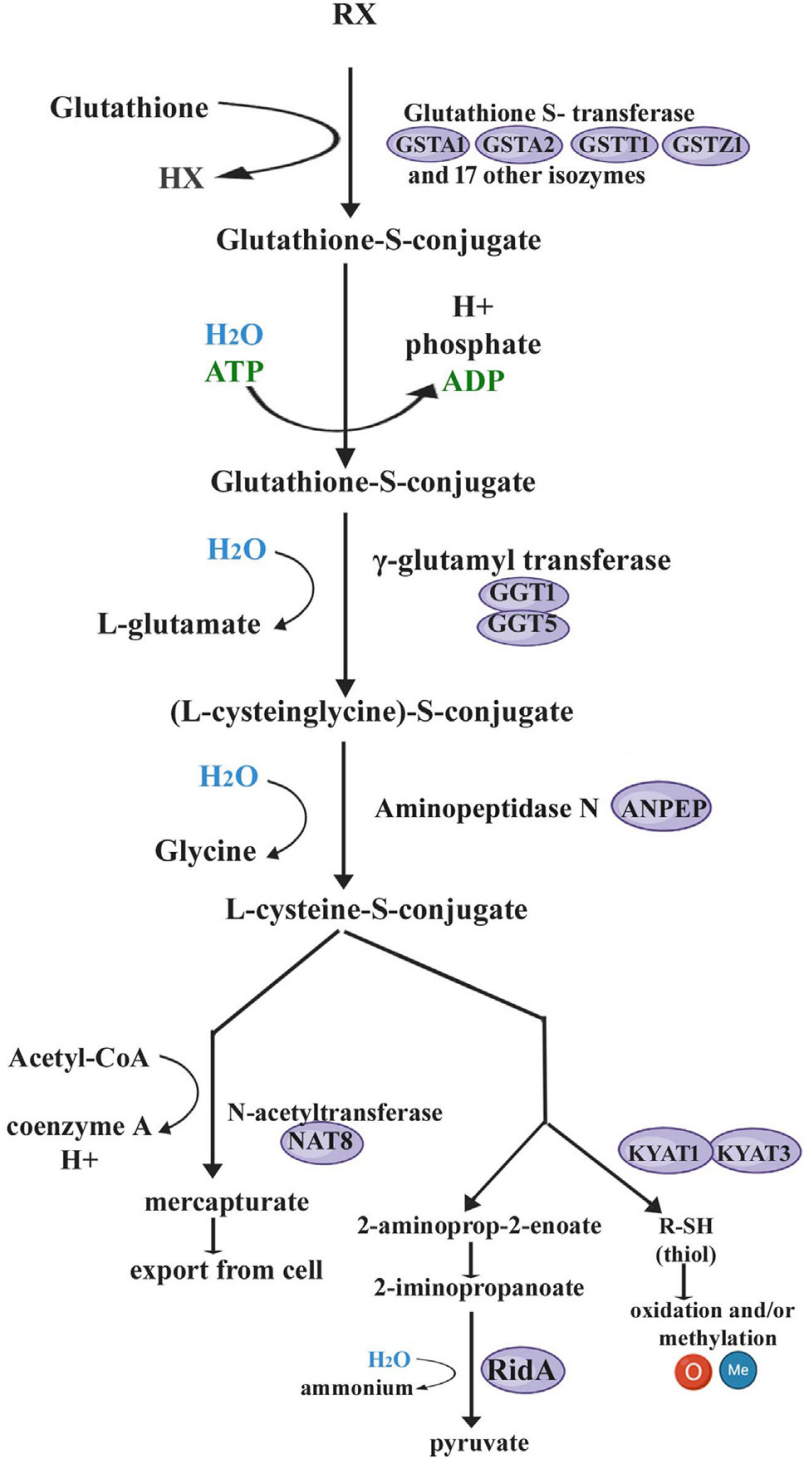
Glutathione S-transferase (GST) mediated detoxification pathway in *Homo sapiens*. R-X is the compound for degradation, where R represents a diverse range of groups, including aliphatic, aromatic, or heterocyclic moieties, while X may denote a sulfate, nitrile, halide group, or even a proton in the reaction. Figure adapted from the MetCyc database^50^ (https://biocyc.org/META/NEW-IMAGE?type=PATHWAY&object=PWY-4061&detail-level=3&ENZORG=TAX-9606&EXP-ONLY=NIL; retrieved 10 February 2024). Distinct GST isozymes exist for rats and mice while a multidrug resistance protein (Abcc1) is present before l-glutamate production in the rat detoxification pathway, indicating a unique aspect of their detoxification mechanism. Mice use folate hydrolase Folh1 and γ-glutamyl hydrolase Ggh instead of GGT1 and GGT5 in humans, while these are missing in the rat pathway. Additionally, while aminopeptidases are common to both mice and humans, they are absent in rats, suggesting variations in protein metabolism. Furthermore, kynurenine aminotransferases (KYAT1 and KYAT3) and N-acetyltransferase (NAT8) are present in humans but not in either rodent species, underscoring significant metabolic differences that could impact toxicological and pharmacological studies.

Numerous enzymes in the cellular defense system of aerobic organisms detoxify reactive oxygen species from metabolic processes, lipid peroxidation, and environmental toxins, and shield cells from macromolecular damage.^26^ The first stages of xenobiotic detoxification are processed by phase-I detoxification enzymes. In the detoxification mechanism, GSH conjugates with electrophilic chemicals, which are then transported out of the cell (Fig. 2). These reactions involve a wide array of foreign chemicals and endogenous reactive intermediates, leading to the formation of various thioether conjugates and thioesters. GSTs catalyze the initial step, followed by metabolization of the GSH-toxin conjugates by degradative enzymes within the cell. The breakdown products are then transported out of the cell and further metabolized, either through acetylation to form mercapturic acids, catalyzed by N-acetyltransferases, or through carbon–sulfur bond breakage mediated by cysteine-S-conjugate β-lyase. These pathways contribute to the enhancement of compound polarity and water solubility, facilitating their excretion from the organism. Additionally, l-cysteine-S-conjugates can undergo alternative enzymatic reactions, including aminotransferase and l-amino acid oxidase activity, offering further avenues for metabolization. Phase-I metabolites are highly apt to form adducts with proteins and nucleic acids because of their electrophilic character.^27^ GSH, recognized as a tripeptide antioxidant, plays a pivotal role in detoxification. GSH synthase facilitates the addition of glycine to a dipeptide to yield GSH, while γ-glutamylcysteine synthetase orchestrates the combination of cysteine and glutamate, both processes entailing adenosine triphosphate hydrolysis.^28^ Predominantly localized within the cytosol, GSH concentrations range from 0.5 to 15 mM, with additional presence in the nucleus and mitochondria, where it regulates cell division and apoptosis.^29^ Its primary function involves scavenging free radicals and capturing reactive oxygen species, which averts permanent damage to cellular components like proteins, lipids, and nucleic acids. Notably, chemicals such as chromium, cadmium, and arsenic rely on GSH metabolism for antioxidant activity, as evidenced by increased *in vitro* toxicities upon depletion of intracellular GSH levels, emphasizing the indispensable role of GSH in detoxification processes facilitated by GSTs.^30^ Natural substrates for GSTs (Table 1) include rac-4-hydroxynonenal, a lipid peroxidation product associated with oxidative stress, and xenobiotics. Other typical substrates for GSTs are pesticides, industrial intermediates, carcinogens, and environmental contaminants.^31^ Environmental carcinogens are ubiquitous in the modern industrial world and pose a hazard to public health. One example is polycyclic aromatic hydrocarbons, which are often found in pharmaceuticals, thermoplastic settings, cigarette smoke, agricultural products, and motor exhaust emissions.^32^

**Table 1:** Natural substrates of major types of GSTs in *Homo sapiens*.

Genes	Substrate	Product	Details	Reference
GSTA1, GSTM1, GSTT1, GSTP1, GSTO1, GSTZ1	Brostallicin and reduced form of glutathione (GSH)	Glutathione conjugated brostallicin	An alpha-chloroamido derivative of the GSH-brostallicin adduct can alkylate DNA and activate brostallicin. Brostallicin is more cytotoxic in cells that overexpress the GST-pi or GST-mu genes and is useful for treating such tumors expressing higher levels of these enzymes.	34
	rac-4-hydroxynonenal + glutathione	S-(4-hydroxy-1-oxononan-3-yl)glutathione	Elimination of the lipid peroxidation product 4-hydroxynonenal, a toxic compound that contributes to numerous diseases.	35
	RX + glutathione	HX + R-S-glutathione	Participates in detoxification	36
			Involved both with bilirubin transport and detoxification of electrophiles	37
			First step in mercapturic acid synthesis	38

Note: These natural substrates of major types of glutathione S-transferases (GSTs) in *Homo sapiens* are listed in the BRENDA database.^33^ RX represents a general placeholder for a reactive xenobiotic compound or drug metabolite.

GSTs play a crucial role in detoxifying the reactive metabolites of methylcholanthrene, a well-known polycyclic aromatic hydrocarbon, by catalyzing its attachment with GSH. This leads to the formation of a highly soluble detoxified complex and facilitates its elimination.^39^ Benzene oxide, another carcinogen, can be detoxified in the body by GSTT1 through its reaction with GSH, ultimately leading to its excretion in urine as S-phenylmercapturic acid.^40^ GSTA1-1 demonstrates high efficiency in catalyzing the detoxification of N-acetoxy-PhIP, a heterocyclic amine derived from food, which is responsible for the development of colorectal cancer.^41^ In the presence of GST and reduced GSH, pesticides diazinon and diazoxon undergo conjugation to form S-(2-isopropyl-4-methylpyrimidin-6-yl)glutathione.^42^ Genipin, a compound from the gardenia fruit has been observed to induce hepatotoxicity, but this effect can be mitigated through the detoxification process facilitated by GSTA1 and GSTA4 enzymes. These enzymes catalyze the conversion of genipin into thiol conjugates, thereby reducing its toxicity and promoting its elimination from the body.^43^ GSTs have also been associated with the development of resistance to microbial antibiotics, insecticides, herbicides, and chemotherapeutic treatments.^44^^,^^45^ In addition to their direct detoxifying function, GSTs also act as inhibitors of the MAP kinase pathway, thereby contributing to the emergence of drug resistance. Chemotherapeutic medicines can be detoxified by them as well, for instance, GSTP1 contributes to chemoresistance in ovarian and other forms of cancer.^46^ Proteomics-based analysis has shown that GSTP1 is overexpressed in cisplatin^47^ and irinotecan^5^ resistant gliomas, fluorouracil (5-FU) and cisplatin resistant gastric cancer cells,^48^ doxorubicin resistant prostate cancer cells,^49^ and adriamycin resistant breast cancer cells.^49^

### Cellular signaling

GSTs also play a significant role in cellular signaling by modulating the activity of protein kinases, regulating the levels of signaling molecules, and interacting with other signaling proteins.^7^ Specifically, GSTs interact with protein kinases, exerting either activating or inhibitory effects on them, thereby influencing cellular processes such as growth, differentiation, and apoptosis.^51^ Additionally, GSTs catalyze the S-glutathionylation of critical cysteine residues within the kinase domain, a post-translational modification that can impact kinase activity, subcellular localization, and downstream signaling cascades.^52^ S-glutathionylation reactions regulate a wide range of physiological functions, such as energy metabolism, glycolysis, immune cell function, cell signaling, calcium homeostasis, and chemotaxis.^53^ All of these responses are essential for monitoring how well cells defend against various environmental assaults.^54^ Furthermore, it has been demonstrated that dysregulated glutathionylation processes contribute to the etiology of numerous human illnesses, such as lung,^55^ neurological,^56^^,^^57^ cardiovascular,^58^ cataract,^59^ and liver disease.^58^

GSTs also perform non-enzymatic functions, effectively inhibiting the activation of downstream targets. For instance, GSTP1, a specific subtype of the GST family, forms a GSTP1-JNK complex, preventing its phosphorylation under normal, non-stressed conditions.^5^ However, in the presence of oxidative stress, the GSTP1-JNK complex dissociates, allowing for JNK activation and GSTP1 dimerization. This highlights the role of GSTP1 as an oxidative stress sensor, adjusting JNK signaling pathways based on the level of reactive oxygen species to promote either cell survival or apoptosis.^60^^,^^61^ Furthermore, GSTs are involved in metabolizing certain signaling molecules like prostaglandins and leukotrienes, thereby influencing their signaling capabilities within the cell.^62^ Additionally, GSTs contribute to the regulation of cellular redox balance by modulating reactive oxygen species and reactive nitrogen species levels through the conjugation of GSH to oxidized molecules.^63^ This enzymatic activity helps prevent oxidative damage and maintain cellular homeostasis. Moreover, GSTs can physically interact with various proteins and structurally diverse non-substrate molecules, including heme, steroids, and bilirubin, thereby impacting signaling pathways and altering their activity.^64^

## Diseases

GSTs are implicated in various diseases due to their crucial role in detoxification and protection against oxidative stress. Dysregulation or genetic variations in GST genes can lead to altered enzyme activity, which may contribute to the development or progression of several diseases, including cancer, neurodegenerative disorders, cardiovascular diseases, and inflammatory conditions (an exhaustive list of diseases due to GST is given in databases like BRENDA (https://www.brenda-enzymes.org/enzyme.php?ecno=2.5.1.18&onlyTable=Disease), DisGeNet (https://disgenet.com/), and PHAROS (https://pharos.nih.gov/targets/) (retrieved 23 September 2024). Many GST variants exhibit similar mechanisms of action across different diseases, mainly through their roles in oxidative stress management.^65^ For instance, both cancer and neurodegenerative diseases often involve increased oxidative damage, where impaired GST function may exacerbate disease progression. Hence, variants such as rs1695 (Ile105Val) affect enzyme activity and have been linked to cancer risk as well as responses to oxidative stress in conditions like Alzheimer's disease.^66^ The association of GST polymorphisms with environmental exposures (*e.g.*, smoking, pollutants) shows similarities across various conditions, suggesting a common pathway where genetic predisposition interacts with external factors. The strength of associations between GST polymorphisms and disease susceptibility can vary significantly. Ethnic variations are a key factor, as certain GST polymorphisms are more prevalent in specific populations. This genetic diversity can lead to differences in how these variants influence disease risk, highlighting the importance of considering genetic background in disease susceptibility studies. An overview of some major classes of diseases due to GST variations is given as follows.

### Cancer

Certain GST variants have been associated with an increased risk of cancer development due to their potential impairment of detoxification processes for carcinogens and reactive oxygen species, thereby promoting DNA damage and oncogenesis.^67^ For instance, genotypes of GSTs have been linked to higher risks of lung,^68^ bladder,^69^ and colorectal cancers.^70^ The GSTA1 ∗A/∗B or ∗B/∗B genotypes have been implicated in an elevated risk of prostate cancer.^71^ Evidence suggests that the combined effect of multiple GST polymorphisms can substantially heighten prostate cancer risk. Individuals with the GSTP1 C haplotype, characterized by the co-occurrence of the GSTP1 Val rs1695 and rs1138272 variants, have a 5.46-fold increased risk of prostate cancer.^72^^,^^73^ The risk escalates to nearly 12-fold in individuals harboring all four risk alleles (GSTM1 active, GSTT1 null, GSTP1 Val rs1695, or GSTP1 Val rs1138272).

The complete gene deletion of GSTM1 and the (GG) genotype of GSTP1 polymorphism (rs1695) have shown significant associations with breast cancer in South Indian^74^ and Jordanian women.^75^ The risk association between GSTM1/GSTT1 deletions and breast cancer development/prognosis has been consistently reported across various studies in Americans, Europeans, and Asians.^76^ Protective effects have been observed in the presence of GSTM1 deletion.^77^ However, the overall association between GST polymorphisms and breast cancer risk remains inconsistent across different studies and populations.^78^ While some studies report significant associations, others do not find any meaningful links, suggesting that genetic background and environmental factors play crucial roles.

The Ile105Val polymorphism of the GSTP1 gene may also genetically predispose individuals to develop skin cancer, particularly malignant melanoma.^79^^,^^80^ The presence of the GSTP1∗C allele has been linked to the development of squamous cell carcinomas.^81^ Variant alleles of GSTP1 have also been correlated with heightened risks of pancreatic cancer.^82^ Moreover, individuals carrying the GSTM1-null genotype face an elevated risk of bladder cancer, a susceptibility that may be exacerbated by factors such as smoking and occupational hazards.^83^

GSTM3 and GSTM1-null genotypes have been implicated in a four-fold risk of larynx cancer.^84^ GSTM3 has been implicated in several cancers, with its protein expression exerting a significant influence on tumor progression or inhibition.^84^, ^85^, ^86^ The GSTM3 rs1055259 variant has been significantly allied with susceptibility to renal cell carcinoma, with evidence suggesting modifications in GSTM3 protein synthesis and reductions in reactive oxygen species activity, thereby affecting progression.^87^ Coric et al have reported that individuals carrying a combination of the GSTM1-null genotype, active GSTT1, low-activity GSTA1, and variant GSTP1 genotypes exhibited a nine-fold increased risk for renal cell carcinoma compared with those with a reference genotype combination.^88^ A pooled meta-analysis by Yang et al^89^ indicates that the influence of genetic background and environmental factors plays a role in renal carcinoma susceptibility. Polymorphisms in GSTM1, GSTT1, and GSTP1 are generally not associated with the development of renal cell carcinoma in certain populations while the active genotypes of GSTM1 and GSTT1 are linked to an increased renal cell carcinoma risk in individuals exposed to pesticides. Additionally, some GST genotypes, particularly GSTT1 and GSTP1, demonstrate positive associations in Asian populations, with the dual null genotype of GSTT1-GSTP1 also indicating a heightened risk.

Moreover, GSTs are involved in multidrug resistance in various cancers by facilitating the detoxification of anti-cancer drugs and interacting with efflux pumps, such as multidrug resistance protein 1 (MRP1) and P-glycoprotein, which can decrease drug efficacy.^10^ GSTP1 expressed in tumor-associated macrophages promotes resistance to the drug adriamycin in breast cancer treatment.^90^ In glioblastoma, GSTM3 overexpression has been linked with poor prognosis and chemotherapy resistance.^91^ In hepatoma, GSTM3 plays a crucial role in reversing radioresistance, indicating its potential therapeutic relevance.^92^ Additionally, GSTs may confer multidrug resistance through non-catalytic mechanisms, such as inhibition of the JNK signaling pathway, protecting tumor cells from apoptosis.^6^

### Neurological diseases

GSTs play a protective role against oxidative stress and protein aggregation in neurodegenerative diseases such as Parkinson's and Alzheimer's disease. Their dysfunction has been implicated in disease pathogenesis^93^ in other neurological disorders like autism spectrum disorder, multiple sclerosis, and amyotrophic lateral sclerosis. GSTs have been found to be overexpressed in epileptic brains.^94^ The null genotypes of GSTT1 and GSTM1, in conjunction with hypertension, are implicated in the pathogenesis of ischemic stroke.^95^ Said et al^96^ have found that the null genotypes for GSTM1 and GSTT1 were the most common in Egyptian children with an autism spectrum disorder. These genotypes may predispose children with autism spectrum disorder to decreased antioxidant status due to reduced GST enzyme activity, potentially leading to impaired detoxification of aluminum. A meta-analysis by Lee et al^97^ has reported that GSTT1 null genotype is associated with multiple sclerosis in Caucasian populations. Fan et al^98^ have reported that the GSTO2 DD genotype is associated with an increased risk of sporadic amyotrophic lateral sclerosis in Chinese men.

GST isoforms mu and pi have been identified as overexpressed in Alzheimer's disease.^99^ Additionally, GSTO1 has been allied with the age of onset for Alzheimer's disease.^100^ E155 deletion in GSTO1 impacts the enzymatic stability and leads to changes in the physiologic role of the enzyme in the brain regions. Apart from this, the GSTP1-1 allelic variant has also been allied with late-onset Alzheimer's disease.^101^ This allelic variant has been suggested to potentially influence the stability of the GSTP1-1/JNK complex and, subsequently, JNK activity, contingent upon the redox status of the cell. In the North Indian population, the deletion of GSTT1 is reported to be significantly associated with Alzheimer's disease.^102^ Moreover, In Italian patients, the GSTM1 null genotype has been identified as a risk factor for late-onset Alzheimer's disease.^103^

A significant correlation exists between the null genotype of GSTT1 and the risk of Parkinson's disease among Caucasians but not in Latinos and Asians.^104^ Chen et al have reported an association between the absence of GSTM1 expression and heightened susceptibility to Parkinson's disease among Caucasians.^105^ In a proteomic study on postmortem samples of Parkinson's disease patients, it was shown that GSTP1 levels were increased.^106^ In the Tunisian population, GSTM1 polymorphism has been found to be significantly associated with the development of tremor as an initial symptom of Parkinson's disease.^107^ GSTM1 and GSTT1 activity is variable in motor impairment among those with the active gene, suggesting a cumulative effect that may not be evident in the early stages of the disease. In the Chilean population, the null mutation in GSTM1 has been associated with Parkinson's disease, particularly among younger individuals.^108^ This supports the hypothesis that GSTM1 may protect astrocytes from the toxic effects of dopamine metabolism, potentially by preventing harmful reductions of aminochrome. In contrast, studies in the Japanese population found no associations between GST polymorphisms and Parkinson's disease,^109^ underscoring the variability in genetic influences across different populations. The presence of the CT variant at codon 114 of GSTP1 is linked to an increased susceptibility to motor neuron disease while GSTP1 polymorphisms are observed to be prevalent in progressive bulbar palsy.^110^ In individuals with progressive myoclonus epilepsy, the presence of a GSTT1-null genotype has been linked to a heightened risk of developing the condition, potentially amplifying susceptibility to oxidative stress.^111^ Similarly, the absence of GSTM1 expression has shown a significant association with epilepsy among Tunisians^112^ but not in the South Indian population. In Serbian children, the combination of the GSTA1∗CC genotype and the GSTT1-null genotype was associated with a higher risk of developing progressive myoclonus epilepsy.^111^ Additionally, GSTs play a role in antiepileptic drug resistance, with elevated levels of GSTP1 in the brain correlating with medication intractability.^113^

### Cardiovascular diseases

GST variants have been allied with an augmented risk of cardiovascular issues.^114^ The absence of the GSTT1 enzyme is identified as an independent, strong predictor of premature vascular morbidity and mortality among individuals with type 2 diabetes.^115^ Furthermore, GSTP1 exhibits a strong correlation with heart failure, suggesting its potential utility as a marker for predicting ventricular function in such cases.^116^ Individuals diagnosed with coronary artery disease exhibit a significant increase in serum GST activity compared with controls.^117^ Furthermore, among coronary artery disease patients, those with concurrent type 2 diabetes mellitus demonstrate even higher levels of serum GST activity, pointing towards marked oxidative stress within this subgroup. Tamer et al have provided evidence indicating that null genotypes of GSTM1 and GSTT1 are linked to a heightened risk of coronary heart disease, especially among individuals who smoke.^118^ Moreover, smokers exhibited more stenosed vessels and higher Duke scores compared with non-smokers. The levels of DNA damage, as assessed by the micronucleus test, were found to be higher in smokers carrying null genotypes of GSTs compared with those with functional genes.^119^ This suggests a potential role of GST-null genotypes in modulating the detoxification of genotoxic atherogens. A meta-analysis by Su et al^120^ found that the GSTM1 null polymorphism is significantly associated with an increased risk of coronary artery disease in both the overall and mixed populations. The GSTP1 null polymorphism was also linked to coronary artery disease risk in the overall population. Additionally, the GSTT1 null polymorphism demonstrated a significant association with coronary artery disease risk across the overall population, as well as in both Caucasian and East Asian populations. A meta-analysis investigating the association between GSTs and coronary heart disease, stratified by smoking status, also unveiled an elevated risk among individuals with null genotypes compared with those with non-null genotypes. This analysis indicated a potential interaction between unfavorable GST genotypes that contributes to the risk of the disease.^121^ Jiang et al^122^ have identified the GSTM3 variant in the people of Taiwan region, China as a novel genetic modifier in Brugada syndrome, a condition associated with an increased risk of sudden cardiac death. Zivkovic et al^123^ have reported that Serbian individuals with the GSTM1 null genotype exhibit a heightened risk of myocardial infarction. In a study conducted in Bangladesh, individuals with the GSTM1 null allele were found to have a 2.5-fold increased risk of experiencing myocardial infarction while those with either the GSTM1 or GSTT1 genotypes exhibited a lower risk.^124^ Furthermore, patients carrying both null genotypes for GSTM1 and GSTT1 showed a 3.5-fold higher risk of myocardial infarction.

### Other diseases

The −69C/T variant (rs3957357) of GSTA1 is allied with an amplified risk of asthma in adults.^125^ Similarly, the G> C variant (rs412543) of GSTM1 and the 16 kb deletion resulting in loss of protein expression are linked to heightened asthma risk, particularly in children.^126^ This risk is further exacerbated when combined with exposure to tobacco smoke.^64^ Additionally, an increased risk of asthma is observed with the GSTO1 haplotype rs156697.^64^ In chronic obstructive pulmonary disease, GSTM1/GSTT1 variants are associated with an elevated risk, particularly in cases of emphysema.^127^ Ding et al^127^ have also found an alliance of null GSTM1 and GSTT1 genotypes with the increased risk of chronic obstructive pulmonary disease. GSTP1 variants like rs1695 and rs1138272, as well as the null variant of GSTT1, also contribute to the risk of asthma and chronic obstructive pulmonary disease.^128^ Individuals deficient in GSTM1 are significantly more likely to develop nonmalignant asbestos-related diseases than those without the deficiency.^129^^,^^130^

Significant associations have been found between deletions of GSTM1 and GSTT1 and the risk of chronic kidney disease in the Chinese population.^131^^,^^132^ Patients with Sjögren's syndrome and systemic lupus erythematosus, who exhibit specific autoantibodies, are found to have a higher occurrence of null alleles for one or more GST enzymes, notably GSTM1.^133^ GSTM1 has also shown significant associations with autoimmune diseases, especially vitiligo and atopic dermatitis.^134^ The GSTM1-positive genotype, as well as the combined “GSTM1-positive/GSTT1-positive" genotype, has been correlated with an elevated risk of developing senile cataracts in an Egyptian cohort.^135^ GST subunit proteins are recognized as a major autoantigen in anti-SLA-positive autoimmune hepatitis.^136^ Heightened prevalence of the GSTM1 null genotype has been observed in patients with lesions exhibiting moderate to severe histological dysplasia.^137^ Among alcoholic individuals, GSTM1 null genetic variant carriers are at an elevated risk of developing alcoholic liver disease,^138^ while studies indicate that in non-alcoholic fatty liver disease, individuals with the Val/Val genotype of GSTP1 and null genotype of GSTT1/GSTM1 null genotype have a higher susceptibility to the disease.^139^ The GSTT1 null genotype has also emerged as a predictor for mortality and case-fatality rates associated with COVID-19.^140^ Moreover, a correlation between the morbidity and mortality of COVID-19 and the Ile105Val polymorphism in GSTP1 has been noted.^141^

## GST inhibitors and their therapeutic implications

GST inhibitors are compounds designed to impede the activity of GSTs, with significant therapeutic implications across various medical conditions. Efforts have been undertaken to create GST inhibitors that can suppress tumor growth and augment the lethal effects of anti-tumor medications.^142^ Additionally, GST inhibitors can sensitize drug-resistant cancer cells to chemotherapy, overcoming resistance mechanisms and improving treatment outcomes. DrugBank lists various GST inhibitors (Table 2) that are in investigational, experimental stage or approved by the US FDA.

**Table 2:** List of various inhibitors of glutathione S-transferase (GST) isoforms.

Gene	Name	Structure	DrugBank ID	Drug group
GSTA1	**Curcumin**		DB11672	Approved, investigational
GSTA2	**Clofibrate**		DB00636	Approved, investigational
	**Etacrynic acid**		DB00903	Approved, investigational
GSTM1	**Curcumin**		DB11672	Approved, investigational
	**Chloroquine**		DB00608	Approved, investigational
GSTP	**Clomipramine**		DB01242	Approved, nutraceutical
	Vitamin E		DB00163	Approved, nutraceutical
	**Exisulind**		DB06246	Investigational
	**Hypericin**		DB13014	Investigational
	**Etacrynic acid**		DB00903	Approved, investigational
	**Alpha-tocopherol succinate**		DB14001	Approved, nutraceutical
	**D-alpha-tocopherol acetate**		DB14002	Approved, nutraceutical
	**Curcumin**		DB11672	Approved, investigational
GSTO1	Vitamin E		DB00163	Approved, nutraceutical
	**Alpha-tocopherol succinate**		DB14001	Approved, nutraceutical
	**D-alpha-Tocopherol acetate**		DB14002	Approved, nutraceutical

Note: The inhibitors of glutathione S-transferase (GST) isoforms are listed in the DrugBank.^143^

GST inhibitors have been investigated as potential neuroprotective agents by mitigating oxidative damage and reducing neuroinflammation.^144^ These inhibitors hold promise for slowing disease progression and preserving cognitive function in affected individuals. In conditions like asthma, inflammatory bowel disease, and rheumatoid arthritis, GST inhibitors may dampen excessive inflammation and alleviate symptoms.^145^ They are categorized according to their structure and binding activity and include molecules that can bind to the G- or H-site of GST proteins, glutathione peptidomimetics, and a number of naturally occurring substances.^146^ Some of these inhibitors are discussed as follows.

### Inhibitors that bind to the G-site

Various isoforms of GSTs exhibit unique G-site structures as revealed by crystallographic studies. This structural insight has been instrumental in the design of inhibitors targeting specific GST subtypes as the G-site recognizes only GSH as a substrate, making it a prototype for developing G-site inhibitors.^5^ However, a major challenge in developing effective G-site inhibitors is the high intracellular concentration of GSH. To overcome this challenge, researchers have devised various strategies, for example, inhibitors targeting GSTP1 were developed by incorporating an electrophilic reactive group that interacts with the thiol group of GSH.^147^ By encircling the thiol group, these inhibitors effectively block GST enzymatic activity. Additionally, to enhance cellular uptake and reduce the need for high inhibitor concentrations, cell membrane-permeable benzene sulfonyl fluoride-type covalent inhibitors have been synthesized. One such benzene sulfonyl fluoride-type inhibitor, derived from the significant GST substrate 1-chloro-2,4-dinitrobenzene, demonstrated promising results in inhibiting GST activity in human non-small-cell lung adenocarcinoma cells.^148^ Washout tests revealed irreversible binding of these inhibitors, resulting in prolonged GST enzyme inactivation. This suggests their potential utility as anti-tumor agents, particularly against tumors with elevated GSTP1 expression.^149^ Furthermore, certain drugs like amitriptyline, commonly prescribed for depression, have been found to bind reversibly to the G-sites of GSTP1 and GSTA1, leading to a significant reduction in their activity.^5^ While this reversible binding may limit their efficacy as GST inhibitors, it highlights the potential of repurposing existing medications for targeting GSTs in various pathological conditions.

### Inhibitors that bind to the H-site

Inhibiting the enzymatic activity of GST proteins can be achieved through various substances that bind to their H-site.^150^ However, developing specific inhibitors for GST subtypes that target the H-site poses a significant challenge due to the diverse range of substrates that can occupy this site.^151^ One notable GST inhibitor, 6-(7-nitro-2,1,3-benzoxadiazol-4-ylthio) hexanol (NBDHEX), exhibits anti-proliferative properties across different cancer cell types.^6^ Structural studies have revealed that NBDHEX interacts with both GSTP1 and GSTM1 in a similar fashion, engaging with their aromatic side chains, particularly Tyr108 in GSTP1 and Tyr115 in GSTM2.^152^ Following the administration of NBDHEX, GSTP1 dissociated from both the GSTP1-JNK and GSTP1-TRAF2 complexes, indicating inhibition of GST activity. Additionally, NBDHEX treatment has led to elevated caspase-dependent apoptosis in various cancer cells, including leukemia cells expressing MDR1. Cancer cells resistant to doxorubicin have exhibited sensitivity to drug treatment upon co-administration of NBDHEX.^153^ Ethacrynic acid, another compound that attaches to the H-site of GST proteins, has also exhibited GSTP1 inhibition.^6^

### Glutathione peptidomimetics

Glutathione peptidomimetics represents another class of compounds that have shown promise as inhibitors of GST enzymes.^154^ These compounds mimic the structure and function of GSH, thereby interfering with GST activity and potentially offering therapeutic benefits. One example is TER199, also known as γ-glutamyl-S-(benzyl)cysteinyl-R-(−)-phenyl glycine diethyl ester; it represents a peptidomimetic analogue of GSH that has been extensively studied. Studies conducted on mouse fibroblast cells treated with TER199 revealed elevated expression levels of γ-glutamyl cysteine synthetase and the multidrug resistance-associated protein, MRP1.^155^ These findings led to the conclusion that inhibition of GSP1 could potentially enhance GSH production and improve the efficacy of chemotherapy medications.^156^ Another peptidomimetic analogue, TLK117, has been extensively investigated in the context of lung fibrosis.^154^ Research indicates that TLK117-mediated suppression of GSTP1 contributes to the inhibition of lung fibrogenesis via JNK signaling pathways. Ezatiostat, also known as TLK199, also serves as a well-known GSTP1 inhibitor and GSH analogue.^157^ In studies involving mouse fibroblast cells, the application of TLK199 disrupted the binding of GSTP1 to JNK and extracellular signal-regulated kinase 2 (ERK2).^158^

### Natural compounds

Natural compounds have emerged as promising inhibitors of GST enzymes, offering potential therapeutic benefits in various diseases, including cancer.^159^ Among the ones targeting the cellular antioxidant system, piperlongumine, derived from *Piper longum* has shown notable GST inhibitory activity.^160^ Studies have revealed that piperlongumine hydrolyzes to hydroxypiperlongumine within cellular environments, which then binds to GSTP1 as a GSH conjugate. Hydroxypiperlongumine interacts with specific residues within the GSTP1 H-site, inhibiting its function and inducing oxidative stress. This mechanism has been associated with diminished tumor growth in the preclinical models of head and neck cancer and pancreatic tumors.^161^ A mouse model with an orthotopic pancreatic tumor showed similar outcomes.^162^ Additionally, curcumin, a naturally occurring compound isolated from *Curcuma longa*, possesses chemopreventive and antioxidant properties. Research suggests that curcumin-induced inactivation of GSTP1 leads to the activation of nuclear factor-κB (NF-κB) and subsequent anti-cancer effects in treated cells.^163^ Flavonoids including baicalin, baicalein, phloridzin, and phloretin have been demonstrated to possess inhibitory effects on human erythrocyte GST^164^ while Majidinia et al have elucidated the suppressive influence of emodin and quercetin on GSTP1, contributing to the mitigation of chemoresistance in cancer cells.^165^ Concurrently, fisetin, a flavonol derived from plants, demonstrated notable capability in diminishing GST expression within colorectal adenocarcinoma cells.^166^ Moreover, oridonin, a tetracyclic diterpenoid extracted from *Rabdosia labtea*, induced markers associated with apoptosis in gemcitabine-resistant PANC-1 pancreatic cancer cells, while concurrently inhibiting the expression of GSTP1.^167^ Natural phenols like resveratrol have also exhibited efficacy in modulating multidrug resistance in tumor cells by reducing GSTs, for example, in doxorubicin-resistant Caco-2 cells.^168^

## Challenges and prospects

The development of inhibitors targeting GSTs is not without challenges and this significantly affects their clinical utility. One primary issue is the presence of conserved binding sites across different GST isoforms, which increases the risk of off-target effects. The inhibitors designed to target one specific isoform may inadvertently affect others, causing unintended interactions or side effects and complicating their therapeutic use. Among the various isoforms impacting chemoresistance,^169^ GSTA1A (−567T, −69C, −52G) plays a role in the resistance to chlorambucil, while GSTA1B (−567G, −69T, −52A) is linked to resistance against busulfan and cyclophosphamide. Additionally, GSTP1C (Val105/Val114) is associated with resistance to paclitaxel and docetaxel, and GSTP1D (Ile105/Val114) impacts resistance to etoposide. Resistance to carmustine is influenced by GSTM1, GSTM3, and GSTT1. Thus, achieving specificity for particular GST variants while minimizing interactions with other isoforms remains challenging due to their structural similarities. While this conservation can be beneficial for targeting multiple pathways in certain contexts, it can also lead to the inhibition of multiple GST isoforms. This inhibition may result in the up-regulation of other isoforms, counteracting the intended therapeutic effects and complicating treatment outcomes.

To address the challenges of developing selective GST inhibitors, structure-based drug design offers a promising solution by utilizing advanced structural biology techniques to engineer isoform-specific inhibitors. While the G site is conserved across GST isoforms, the H site and other regions may vary, providing opportunities to create more selective inhibitors that minimize off-target effects. High-throughput screening could come in handy to identify selective compounds. Fragment-based drug design using smaller, more targeted molecules could also help in designing focused inhibitors. Allosteric site modulators can provide an alternative mechanism for inhibition without affecting the active site of other isoforms. Engineering bioconjugates that link inhibitors to targeting moieties, such as antibodies or peptides, could effectively direct these inhibitors to specific tissues or cells that express the target GST isoform. Combining GST inhibitors with other therapeutic agents targeting different cellular pathways may further enhance efficacy and reduce dependence on a single target. This combination therapy approach holds potential for overcoming drug resistance and improving treatment outcomes.

Population-specific polymorphisms in GST genes pose significant challenges by causing variations in protein expression and function. These variations can impact enzymatic activity and binding affinities of the target isoform across different cohorts, raising concerns about the efficacy of inhibitors tailored to specific genetic profiles. Furthermore, polymorphisms in the same disease across diverse populations necessitate extensive clinical trials to account for genetic diversity, highlighting the need for a personalized approach to drug development. Addressing these challenges will require focused research efforts, advancements in pharmacogenomics, and personalized medicine strategies to improve therapeutic outcomes and ensure the safety of GST inhibitors across various demographic groups. Conducting clinical trials that encompass a wide range of ethnic and demographic backgrounds is essential for gathering data on the efficacy and safety of GST inhibitors in different genetic contexts. Additionally, challenges related to diet, lifestyle patterns, and metabolism may require further consideration. Incorporating excipients to enhance the solubility and stability of GST inhibitors could improve bioavailability and efficacy among populations with varying metabolic rates. Utilizing advanced drug delivery systems, such as nanoparticles, may also enhance the targeting and absorption of GST inhibitors, potentially addressing differences in absorption rates across diverse populations.

Concurrently, pharmacokinetic optimization strategies are vital for improving the bioavailability, tissue distribution, and overall therapeutic efficacy of GST inhibitors. Innovative solutions such as prodrug strategies and targeted drug delivery systems can enhance drug stability and minimize systemic toxicity, maximizing the therapeutic potential of GST inhibitors. Future prospects lie in the continued refinement of these technologies to enhance specificity and reduce off-target effects, ultimately leading to more effective treatments for diseases influenced by GST isoforms.

## Conclusion

GSTs are pivotal in the detoxification processes that mitigate the impact of harmful metabolites and toxins in the human body. Their ability to conjugate harmful substances, including reactive oxygen species and lipid peroxidation products, underscores their protective role in cellular health. Polymorphisms in GST genes are associated with an increased risk of several diseases, including cancer, respiratory disorders, liver diseases, and autoimmune conditions. GST inhibitors have been developed to target specific isoforms of these enzymes for disease treatment. They also significantly influence drug metabolism, affecting the pharmacokinetics of various therapeutic agents. This dual functionality is particularly relevant in oncology, where GST overexpression is often linked to chemoresistance. Researchers are thus focusing on developing inhibitors that target drug-resistant isoforms, enhancing the effectiveness of existing chemotherapeutics. Additionally, understanding GST genetic polymorphisms across diverse populations can enable personalized treatment approaches. Continued exploration of GST inhibitors and their mechanisms is essential for advancing treatments and improving the management of health challenges.

## Author contributions

The author conceived, designed, conducted, wrote, and edited the review.

## Funding

The Researchers would like to thank the Deanship of Graduate Studies and Scientific Research at 10.13039/501100007414Qassim University for financial support (QU-APC-2024-9/1).

## CRediT authorship contribution statement

**Sulaiman Mohammad Alnasser:** Conceptualization, Data curation, Formal analysis, Funding acquisition, Investigation, Methodology, Project administration, Resources, Software, Supervision, Validation, Visualization, Writing – original draft, Writing – review & editing.

## Conflict of interests

The author declared no conflict of interests.
